# Bis[2-(2-pyridylmethyl­eneamino)benzene­sulfonato-κ^3^ *N*,*N*′,*O*]cobalt(II) dihydrate

**DOI:** 10.1107/S1600536809043918

**Published:** 2009-10-31

**Authors:** Xue-Ren Huang, Miao Ou-Yang, Ge-Ge Yang, Xiu-Jin Meng, Yi-Min Jiang

**Affiliations:** a College of Chemistry and Chemical Engineering, Guangxi Normal University, Guilin 541004, People’s Republic of China; b Key Laboratory of New Processing Technology for Nonferrous Metals & Materials, Ministry of Education, Guilin University of Technology, Guilin 541004, People’s Republic of China

## Abstract

The title complex, [Co(C_12_H_9_N_2_O_3_S)_2_]·2H_2_O, has site symmetry 2 with the Co^II^ cation located on a twofold rotation axis. Two tridentate 2-(2-pyridylmethyl­eneamino)benzene­sulfonate (paba) ligands chelate to the Co^II^ cation in a distorted octa­hedral geometry. The pyridine and benzene rings in the paba ligand are oriented at a dihedral angle of 42.86 (13)°. Inter­molecular O—H⋯O and C—H⋯O hydrogen bonding is present in the crystal structure.

## Related literature

For general background to the coordination chemistry of the sulfonate ligands, see: Jiang *et al.* (2006 ▶). For the isostructural Zn and Cd complexes, see: Cai *et al.* (2008 ▶); Ou-Yang *et al.* (2008 ▶). For the synthesis, see: Casella & Gullotti (1986 ▶).
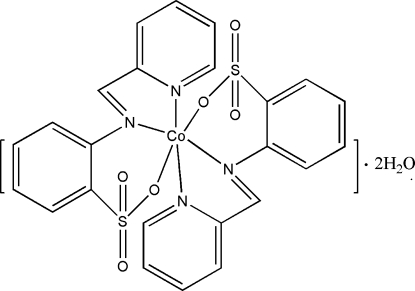


## Experimental

### 

#### Crystal data

[Co(C_12_H_9_N_2_O_3_S)_2_]·2H_2_O
*M* *_r_* = 617.53
Orthorhombic, 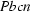

*a* = 19.636 (2) Å
*b* = 8.0973 (8) Å
*c* = 16.2819 (16) Å
*V* = 2588.8 (4) Å^3^
*Z* = 4
Mo *K*α radiation
μ = 0.88 mm^−1^
*T* = 291 K
0.31 × 0.25 × 0.07 mm

#### Data collection

Bruker SMART CCD area-detector diffractometer
Absorption correction: multi-scan (*SADABS*; Sheldrick, 1996 ▶) *T*_min_ = 0.771, *T*_max_ = 0.939
17970 measured reflections
2410 independent reflections
1960 reflections with *I* > 2σ(*I*)
*R*_int_ = 0.039

#### Refinement

*R*[*F* ^2^ > 2σ(*F* ^2^)] = 0.032
*wR*(*F* ^2^) = 0.089
*S* = 1.02
2410 reflections
177 parameters
H-atom parameters constrained
Δρ_max_ = 0.44 e Å^−3^
Δρ_min_ = −0.46 e Å^−3^

### 

Data collection: *SMART* (Bruker, 2004 ▶); cell refinement: *SAINT* (Bruker, 2004 ▶); data reduction: *SAINT*; program(s) used to solve structure: *SHELXTL* (Sheldrick, 2008 ▶); program(s) used to refine structure: *SHELXTL*; molecular graphics: *SHELXTL*; software used to prepare material for publication: *SHELXTL*.

## Supplementary Material

Crystal structure: contains datablocks I, global. DOI: 10.1107/S1600536809043918/xu2642sup1.cif

Structure factors: contains datablocks I. DOI: 10.1107/S1600536809043918/xu2642Isup2.hkl

Additional supplementary materials:  crystallographic information; 3D view; checkCIF report

## Figures and Tables

**Table 1:** Selected bond lengths (Å)

Co1—O3	2.1029 (16)
Co1—N1	2.1863 (18)
Co1—N2	2.147 (2)

**Table 2:** Hydrogen-bond geometry (Å, °)

*D*—H⋯*A*	*D*—H	H⋯*A*	*D*⋯*A*	*D*—H⋯*A*
O1—H1*W*⋯O4^i^	0.85	2.16	3.009 (3)	179
O1—H2*W*⋯O2	0.84	2.15	2.867 (3)	143
C7—H7⋯O1^ii^	0.93	2.56	3.425 (3)	154
C11—H11⋯O4^iii^	0.93	2.46	3.389 (3)	172

Symmetry codes: (i) 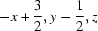; (ii) 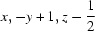; (iii) 
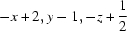
.

**Fig. 1.**
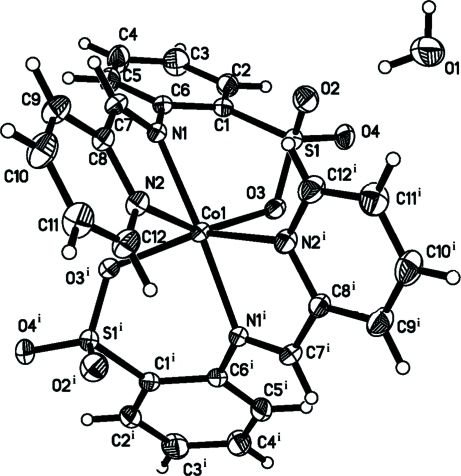
The molecular structure of the complex, showing the atom-numbering scheme. Symmetry code: -x + 2, y, -z + 1/2.

## supplementary crystallographic information

### Comment

The design of supermolecular coordination complexes in which both coordination bonds and hydrogen bonds take part in the self-assembly chemistry have recently generated increasing interest. Our group have focused on the exploration of the coordination chemistry of the sulfonate ligands (Jiang *et al.*., 2006). We report here the structure of the title complex (Fig. 1).

The Co^II^ complex is isostructural with [Zn(Paba)_2_].2H_2_O and [Cd(Paba)_2_].2H_2_O whose structure has been described in detail (Cai *et al.*., 2008; Ou-Yang *et al.*., 2008). The Co(II) atom lies on the twofold rotation axis and is coordinated by pyridine N, imine N and sulfonate O atoms from two paba^-^ ligands with a distorted octahedral geometry (Table 1). This structure is similar to complexes with N,N',O-tridentate donor ligands (Casella *et al.*., 1986). The O—H···O and C—H···O hydrogen bonding (Table 2) is present in the crystal structure.

### Experimental

The potassium salt of 2-(pyridylmethyl)imine-2-benzenesulfonic acid (pabaK) was synthesized according to the literature method (Casella & Gullotti, 1986). To prepare the title complex, pabaK (1 mmol, 0.30 g) was dissolved in methanol (10 ml) at 333 k and an aqueous solution (10 ml) containing Co(AcO)_2_.4H_2_O (0.5 mmol, 0.125 g) was added. The mixture was stirred at 333 K for 4 h, then cooled to room temperature and filtered. Red crystals suitable for X-ray diffraction were obtained by slowly evaporation over several days, with a yield of 60%. Elemental analysis, found (%): C: 46.59; H: 4.04; N: 9.11; S: 10.25, calc (%): C: 46.64; H: 3.56; N: 9.07; S: 10.36.

### Refinement

H atoms bonded to C atoms were positioned geometrically with the C—H distance of 0.93 A, and treated as riding atoms, with *U*_iso_(H) = 1.2U_eq_(C). Water H atoms were placed in a difference Fourier map and refined as riding in as-found relative positions with *U*_iso_(H) = 1.5U_eq_(O).

### Crystal data

**Table:**

[Co(C_12_H_9_N_2_O_3_S)_2_]·2H_2_O	*F*(000) = 1268
*M**_r_* = 617.53	*D*_x_ = 1.584 Mg m^−^^3^
Orthorhombic, *P**b**c**n*	Mo *K*α radiation, λ = 0.71073 Å
Hall symbol: -P 2n 2ab	Cell parameters from 4317 reflections
*a* = 19.636 (2) Å	θ = 2.5–24.7°
*b* = 8.0973 (8) Å	µ = 0.88 mm^−^^1^
*c* = 16.2819 (16) Å	*T* = 291 K
*V* = 2588.8 (4) Å^3^	Block, red
*Z* = 4	0.31 × 0.25 × 0.07 mm

### Data collection

**Table:**

Bruker SMART CCD area-detector diffractometer	2410 independent reflections
Radiation source: fine-focus sealed tube	1960 reflections with *I* > 2σ(*I*)
graphite	*R*_int_ = 0.039
φ and ω scans	θ_max_ = 25.5°, θ_min_ = 2.5°
Absorption correction: multi-scan (*SADABS*; Sheldrick, 1996)	*h* = −23→23
*T*_min_ = 0.771, *T*_max_ = 0.939	*k* = −9→9
17970 measured reflections	*l* = −19→19

### Refinement

**Table:**

Refinement on *F*^2^	Primary atom site location: structure-invariant direct methods
Least-squares matrix: full	Secondary atom site location: difference Fourier map
*R*[*F*^2^ > 2σ(*F*^2^)] = 0.032	Hydrogen site location: inferred from neighbouring sites
*wR*(*F*^2^) = 0.089	H-atom parameters constrained
*S* = 1.02	*w* = 1/[σ^2^(*F*_o_^2^) + (0.0467*P*)^2^ + 1.5384*P*] where *P* = (*F*_o_^2^ + 2*F*_c_^2^)/3
2410 reflections	(Δ/σ)_max_ = 0.001
177 parameters	Δρ_max_ = 0.44 e Å^−^^3^
0 restraints	Δρ_min_ = −0.46 e Å^−^^3^

### Special details

**Table:**

Geometry. All e.s.d.'s (except the e.s.d. in the dihedral angle between two l.s. planes) are estimated using the full covariance matrix. The cell e.s.d.'s are taken into account individually in the estimation of e.s.d.'s in distances, angles and torsion angles; correlations between e.s.d.'s in cell parameters are only used when they are defined by crystal symmetry. An approximate (isotropic) treatment of cell e.s.d.'s is used for estimating e.s.d.'s involving l. s. planes.
Refinement. Refinement of *F*^2^ against ALL reflections. The weighted *R*-factor *wR* and goodness of fit *S* are based on *F*^2^, conventional *R*-factors *R* are based on *F*, with *F* set to zero for negative *F*^2^. The threshold expression of *F*^2^ > σ(*F*^2^) is used only for calculating *R*-factors(gt) *etc*. and is not relevant to the choice of reflections for refinement. *R*-factors based on *F*^2^ are statistically about twice as large as those based on *F*, and *R*- factors based on ALL data will be even larger.

### Fractional atomic coordinates and isotropic or equivalent isotropic displacement parameters (Å^2^)

**Table:**

	*x*	*y*	*z*	*U*_iso_*/*U*_eq_	
Co1	1.0000	0.68407 (5)	0.2500	0.02746 (14)	
S1	0.87615 (3)	0.82456 (8)	0.34750 (3)	0.03191 (17)	
O1	0.79780 (13)	0.4090 (4)	0.44814 (16)	0.1166 (13)	
H1W	0.7550	0.4121	0.4400	0.175*	
H2W	0.8188	0.4425	0.4064	0.175*	
O2	0.84711 (9)	0.6617 (2)	0.34011 (11)	0.0477 (5)	
O3	0.95125 (8)	0.8220 (2)	0.34231 (9)	0.0350 (4)	
O4	0.85336 (9)	0.9170 (2)	0.41814 (10)	0.0438 (4)	
N1	0.90902 (9)	0.7307 (2)	0.17616 (11)	0.0297 (4)	
N2	0.99827 (10)	0.4858 (3)	0.16249 (12)	0.0335 (5)	
C1	0.85084 (11)	0.9367 (3)	0.25933 (13)	0.0310 (5)	
C2	0.81355 (13)	1.0800 (3)	0.26678 (15)	0.0413 (6)	
H2	0.8016	1.1189	0.3186	0.050*	
C3	0.79387 (15)	1.1660 (4)	0.19754 (18)	0.0534 (8)	
H3	0.7691	1.2634	0.2027	0.064*	
C4	0.81090 (14)	1.1074 (4)	0.12064 (16)	0.0538 (8)	
H4	0.7974	1.1653	0.0741	0.065*	
C5	0.84796 (13)	0.9631 (4)	0.11249 (15)	0.0444 (7)	
H5	0.8593	0.9246	0.0604	0.053*	
C6	0.86829 (11)	0.8756 (3)	0.18134 (14)	0.0309 (5)	
C7	0.89905 (12)	0.6270 (3)	0.11848 (14)	0.0362 (6)	
H7	0.8624	0.6399	0.0828	0.043*	
C8	0.94554 (12)	0.4883 (3)	0.10895 (13)	0.0330 (5)	
C9	0.93760 (15)	0.3705 (3)	0.04869 (16)	0.0469 (7)	
H9	0.9008	0.3749	0.0128	0.056*	
C10	0.98533 (16)	0.2455 (4)	0.04253 (18)	0.0534 (7)	
H10	0.9808	0.1639	0.0027	0.064*	
C11	1.03915 (16)	0.2432 (3)	0.09565 (18)	0.0503 (7)	
H11	1.0722	0.1612	0.0919	0.060*	
C12	1.04376 (14)	0.3648 (3)	0.15512 (16)	0.0428 (6)	
H12	1.0802	0.3618	0.1915	0.051*	

### Atomic displacement parameters (Å^2^)

**Table:**

	*U*^11^	*U*^22^	*U*^33^	*U*^12^	*U*^13^	*U*^23^
Co1	0.0260 (2)	0.0333 (3)	0.0231 (2)	0.000	−0.00434 (16)	0.000
S1	0.0305 (3)	0.0415 (4)	0.0238 (3)	−0.0001 (3)	0.0004 (2)	0.0016 (2)
O1	0.0579 (15)	0.193 (3)	0.099 (2)	−0.0322 (18)	−0.0192 (14)	0.079 (2)
O2	0.0511 (11)	0.0469 (11)	0.0452 (11)	−0.0111 (9)	−0.0011 (9)	0.0076 (9)
O3	0.0292 (8)	0.0485 (10)	0.0272 (8)	0.0047 (7)	−0.0040 (7)	−0.0038 (7)
O4	0.0425 (10)	0.0625 (12)	0.0263 (9)	0.0069 (9)	0.0045 (7)	−0.0037 (8)
N1	0.0253 (9)	0.0408 (11)	0.0231 (10)	0.0000 (8)	−0.0006 (7)	−0.0016 (9)
N2	0.0383 (11)	0.0336 (11)	0.0287 (10)	0.0019 (9)	−0.0042 (8)	−0.0010 (8)
C1	0.0238 (11)	0.0400 (13)	0.0293 (12)	−0.0001 (10)	−0.0006 (9)	0.0029 (10)
C2	0.0354 (14)	0.0526 (16)	0.0357 (13)	0.0096 (12)	0.0024 (11)	−0.0009 (12)
C3	0.0490 (16)	0.0553 (18)	0.0560 (18)	0.0229 (14)	0.0011 (14)	0.0072 (14)
C4	0.0512 (17)	0.072 (2)	0.0377 (15)	0.0214 (15)	−0.0016 (12)	0.0139 (14)
C5	0.0389 (14)	0.0639 (19)	0.0305 (13)	0.0118 (13)	0.0013 (11)	0.0054 (12)
C6	0.0229 (11)	0.0433 (13)	0.0266 (11)	0.0005 (10)	−0.0010 (9)	0.0015 (11)
C7	0.0301 (12)	0.0503 (15)	0.0282 (12)	−0.0005 (11)	−0.0073 (10)	−0.0023 (11)
C8	0.0342 (12)	0.0382 (13)	0.0265 (12)	−0.0024 (11)	−0.0032 (10)	−0.0016 (10)
C9	0.0526 (16)	0.0487 (16)	0.0395 (15)	−0.0023 (13)	−0.0109 (12)	−0.0098 (13)
C10	0.073 (2)	0.0435 (16)	0.0433 (17)	0.0039 (15)	−0.0087 (15)	−0.0127 (13)
C11	0.0646 (19)	0.0376 (15)	0.0488 (17)	0.0134 (14)	−0.0012 (14)	−0.0036 (13)
C12	0.0492 (16)	0.0410 (15)	0.0381 (14)	0.0070 (12)	−0.0085 (12)	−0.0004 (12)

### Geometric parameters (Å, °)

**Table:**

Co1—O3	2.1029 (16)	C2—C3	1.380 (4)
Co1—O3^i^	2.1029 (16)	C2—H2	0.9300
Co1—N1^i^	2.1862 (18)	C3—C4	1.380 (4)
Co1—N1	2.1863 (18)	C3—H3	0.9300
Co1—N2	2.147 (2)	C4—C5	1.383 (4)
Co1—N2^i^	2.147 (2)	C4—H4	0.9300
S1—O2	1.4421 (18)	C5—C6	1.385 (3)
S1—O4	1.4433 (17)	C5—H5	0.9300
S1—O3	1.4773 (17)	C7—C8	1.456 (3)
S1—C1	1.770 (2)	C7—H7	0.9300
O1—H1W	0.8502	C8—C9	1.378 (3)
O1—H2W	0.8402	C9—C10	1.383 (4)
N1—C7	1.275 (3)	C9—H9	0.9300
N1—C6	1.422 (3)	C10—C11	1.366 (4)
N2—C12	1.331 (3)	C10—H10	0.9300
N2—C8	1.354 (3)	C11—C12	1.384 (4)
C1—C2	1.377 (3)	C11—H11	0.9300
C1—C6	1.406 (3)	C12—H12	0.9300
			
O3—Co1—O3^i^	115.85 (9)	C1—C2—H2	119.9
O3—Co1—N2	149.80 (7)	C3—C2—H2	119.9
O3^i^—Co1—N2	85.98 (7)	C2—C3—C4	120.0 (3)
O3—Co1—N2^i^	85.98 (7)	C2—C3—H3	120.0
O3^i^—Co1—N2^i^	149.80 (7)	C4—C3—H3	120.0
N2—Co1—N2^i^	83.20 (11)	C3—C4—C5	120.3 (2)
O3—Co1—N1^i^	83.51 (6)	C3—C4—H4	119.8
O3^i^—Co1—N1^i^	85.95 (6)	C5—C4—H4	119.8
N2—Co1—N1^i^	120.48 (7)	C4—C5—C6	120.4 (2)
N2^i^—Co1—N1^i^	75.60 (7)	C4—C5—H5	119.8
O3—Co1—N1	85.95 (6)	C6—C5—H5	119.8
O3^i^—Co1—N1	83.51 (6)	C5—C6—C1	118.7 (2)
N2—Co1—N1	75.60 (7)	C5—C6—N1	122.4 (2)
N2^i^—Co1—N1	120.48 (7)	C1—C6—N1	118.78 (19)
N1^i^—Co1—N1	160.09 (11)	N1—C7—C8	119.4 (2)
O2—S1—O4	114.72 (11)	N1—C7—H7	120.3
O2—S1—O3	112.14 (11)	C8—C7—H7	120.3
O4—S1—O3	111.25 (10)	N2—C8—C9	122.3 (2)
O2—S1—C1	106.90 (11)	N2—C8—C7	115.0 (2)
O4—S1—C1	107.06 (11)	C9—C8—C7	122.6 (2)
O3—S1—C1	103.96 (10)	C8—C9—C10	118.8 (2)
H1W—O1—H2W	110.5	C8—C9—H9	120.6
S1—O3—Co1	120.19 (9)	C10—C9—H9	120.6
C7—N1—C6	120.03 (19)	C11—C10—C9	119.2 (3)
C7—N1—Co1	114.63 (16)	C11—C10—H10	120.4
C6—N1—Co1	124.72 (14)	C9—C10—H10	120.4
C12—N2—C8	117.8 (2)	C10—C11—C12	118.9 (3)
C12—N2—Co1	126.85 (16)	C10—C11—H11	120.5
C8—N2—Co1	115.36 (16)	C12—C11—H11	120.5
C2—C1—C6	120.4 (2)	N2—C12—C11	122.9 (2)
C2—C1—S1	120.67 (18)	N2—C12—H12	118.6
C6—C1—S1	118.91 (18)	C11—C12—H12	118.6
C1—C2—C3	120.1 (2)		

Symmetry codes: (i) −*x*+2, *y*, −*z*+1/2.

### Hydrogen-bond geometry (Å, °)

**Table:**

*D*—H···*A*	*D*—H	H···*A*	*D*···*A*	*D*—H···*A*
O1—H1W···O4^ii^	0.85	2.16	3.009 (3)	179
O1—H2W···O2	0.84	2.15	2.867 (3)	143
C7—H7···O1^iii^	0.93	2.56	3.425 (3)	154
C11—H11···O4^iv^	0.93	2.46	3.389 (3)	172

Symmetry codes: (ii) −*x*+3/2, *y*−1/2, *z*; (iii) *x*, −*y*+1, *z*−1/2; (iv) −*x*+2, *y*−1, −*z*+1/2.
